# Porphyria: a case report

**DOI:** 10.1186/s13256-022-03708-w

**Published:** 2022-12-28

**Authors:** Sujata Baidya, Pratibha Kandel, Smrity Rajkarnikar, Anuradha Kadel, Apeksha Niraula, Raju Kumar Dubey, Machhindra Lamichhane, Mithileshwer Raut, Aseem Bhattarai, Eans Tara Tuladhar, Vijay Kumar Sharma

**Affiliations:** 1 Department of Clinical Biochemistry, Institute of Medicine, Maharajgunj Medical Campus, Kathmandu, Nepal; 2 Department of Paediatrics, Institute of Medicine, Maharajgunj Medical Campus, Kathmandu, Nepal

**Keywords:** Acute intermittent porphyria, Adolescent, Abdominal pain, Hyponatremia, Constipation

## Abstract

**Background:**

Prompt diagnosis of metabolic disorders in a resource-limited country like Nepal is daunting. Acute intermittent porphyria is a rare but common hepatic porphyria mostly seen in females of the reproductive age group. As its incidence is quite uncommon, conjectures about porphyria diagnosis are often duped into a diagnostic conundrum.

**Case presentation:**

Here we unravel a case of a 15-year-old Hindu Nepalese girl distraught by the myriad of symptoms in the setting of severe abdominal pain accompanied by constipation and limb pain as the chief complaints. She presented with acute severe hypertension with marked persistent hyponatremia (up to 109 mEq/L). Despite conservative management of hypertension and electrolytes, unresolved electrolyte imbalance led us to the speculation of disturbance in the renin–angiotensin–aldosterone system. Due to her exacerbating neurovisceral status, she also required intensive care during the disease course. After thorough investigations and exemption of presumed provisional diagnoses, based on sustained symptomatic presentation, the clinical suspicion was driven towards a diagnosis of porphyria-related disorders. Positive Watson-Schwartz test substantiated the diagnosis of acute intermittent porphyria. Her symptoms gradually abated after the consumption of high carbohydrate diets.

**Conclusion:**

This case highlights the baffling amalgamation of symptoms that simulate common diseases of concern yet are buried in the realm of porphyric disorders. Porphyria can be diagnosed using simple screening tools and timely treatment can diminish serious consequences.

**Supplementary Information:**

The online version contains supplementary material available at https://doi.org/10.1186/s13256-022-03708-w.

## Background

Porphyrias are rare metabolic disorders, which comprise a group of inherited and acquired disorders wherein there is a partial or complete deficiency of enzymes in the heme biosynthetic pathway [1]. Disturbance in the heme synthetic pathway causes the intermediates in these steps to accumulate. Marked as a “little imitator”, porphyria is masqueraded by a myriad of clinical manifestations ranging from neurovisceral symptoms to various dermatological manifestations [2]. Early intermediates are porphyrin precursors that mostly parade neurological symptoms whereas late intermediates are porphyrins that are photosensitive and manifest dermatological symptoms [3]. Acute intermittent porphyria (AIP) is the most common form of inherited hepatic porphyrias seen in women of the reproductive age group. Factors that incite acute attacks of porphyria include alcohol ingestion, a low-carbohydrate diet, fasting, menstruation, infection, surgical procedures, and a variety of porphyrinogenic drugs [4]. Preliminary screening of AIP is done by demonstrating markedly elevated urinary porphobilinogen levels during an acute attack followed by genetic analysis of HMBS gene mutation for confirmation [5].

Still, only a handful of porphyria cases have been reported in Nepal so far in the medical literature. Here we present a case of porphyria presenting severe pain in the abdomen with intermittent exacerbation, constipation, and hypertension as the only presentation. After a thorough investigation ruling out the differential diagnosis, a final diagnosis of acute intermittent porphyria was made based on laboratory findings.

## Case presentation

A 15-year-old Hindu Nepalese schoolgirl presented to the Paediatric Emergency Department at Tribhuvan University Teaching Hospital with the complaint of severe abdominal pain for the last few days accompanied by headache, nausea, vomiting, limb pain, and constipation.

The patient had similar episodes of acute abdominal pain twice before with no definite diagnosis explaining her pain. Four months ago, she had her first episode of abdominal pain, for which she was admitted to a private tertiary care hospital in Biratnagar for a presumed Intestinal Obstruction due to fecal impaction.

In the second trivia of pain, she was again admitted to another private tertiary care hospital in Kathmandu for abdominal pain, constipation, pain in limbs, and hypertension. According to the informant, the patient was essentially well 4 days before admission. The patient stated that the abdominal pain was acute at the onset, originating at the left hypochondrium, which was non-radiating, colicky, and burning in nature at the same time with waxing and waning intensity. She also complained of multiple episodes of vomiting which was non-bilious and non-bloody in character, accompanied by loss of appetite. Also, she complained of intermittent constipation for the past 3 months and a history of passing stool only once a week. There was no history of fever, cough, headache, weight loss, progressive paleness, swelling of the body, haematuria, and loss of consciousness. During her stay at the hospital, she was admitted to PICU for 3 days. Labetalol infusion was started for acute severe hypertension. She also had episodes of generalized tonic–clonic seizure, which was managed with anti-epileptics. In the laboratory test, the patient was found to have hyponatremia. A presumed diagnosis of Gitelman Syndrome with chronic constipation and refractory hypokalemia was made. Apart from these, the patient did not have any other significant past medical history. She was then referred to our center for further management.

She was admitted to Tribhuvan University Teaching Hospital (TUTH) Paediatric Emergency on 20th August 2022. On examination, the patient was alert and well-oriented and she had no pallor, icterus, cyanosis, lymphadenopathy, or edema. There was no blistering, scarring, or erythema on the skin either. Detailed family history revealed no significant inherited disorders. Likewise, there was no relevant past surgical, social or psycho-social history recorded in our case. Her menstrual history elucidated she had oligomenorrhea but no significant precipitation of symptoms related to her current illness.

On admission, her vitals on examination were; blood pressure (180/100 mmHg), respiratory rate (20 bpm), SpO2 (92%), pulse rate (100 bpm), and normal respiratory and cardiovascular system examination. Per abdominal examination, a mildly tender abdomen was noted, while no organomegaly and renal bruits were noted. CNS examination showed the patient was normal with a Glasgow Coma Scale (GCS) of 15/15. Comprehensive blood count was within the normal limits at the time of admission except for a mild anemic picture with a hemoglobin of 10.9 g/dl.

For the management of hypertension, amlodipine was started. Polyethylene glycol was given for constipation. 3% NaCl infusion followed by oral salt replacement was started to correct sodium levels. However, persistent hyponatremia (up to 109 mEq/L) was the area of concern along with hypomagnesemia (ranging from 1.1 to 2.4 mg/dl). It raised the speculation of disturbance in the renin–angiotensin–aldosterone system. After laboratory analysis, serum aldosterone was 45.94 ng/dl (3.1–35.1 ng/dl in the upright position), direct renin 161.73 µIU/ml (4.67–47.60 µIU/ml in the upright position), and aldosterone renin ratio (ARR) was 0.28. Urinary sodium output was also elevated (169 mEq/L). So unilateral renal artery stenosis (RAS) was suspected. To confirm RAS, abdominal doppler and pelvic CT investigations were requested. The CT and Doppler report was normal, excluding the diagnosis of renal artery stenosis.

Similarly, arterial blood gas (ABG) analysis was normal, which was against the metabolic alkalosis seen in Gitelman syndrome. To rule out an autoimmune cause, an antinuclear antibody (ANA) was sent; however, it also came out to be negative (Additional file 1).

**Fig. 1:**
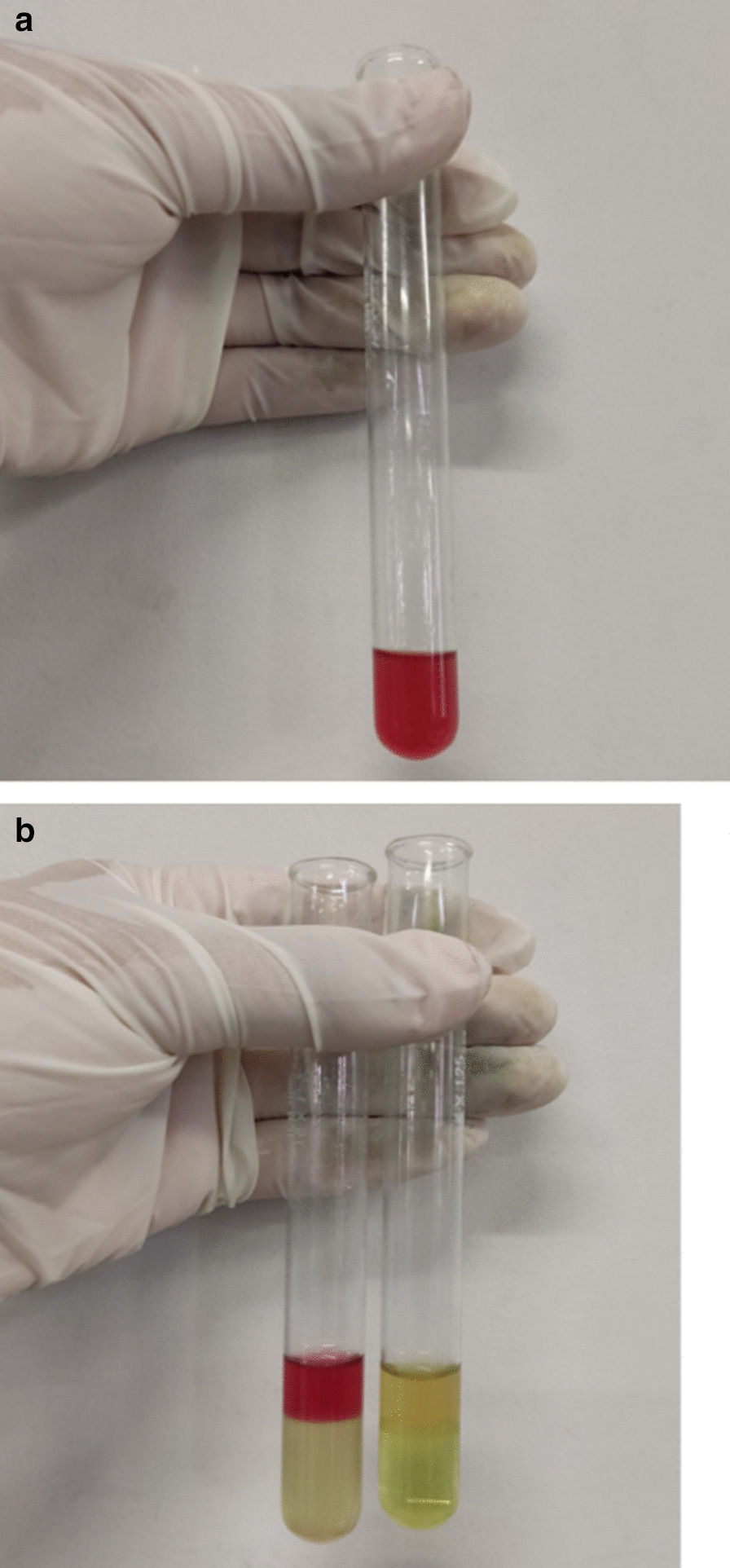
Watson-Schwartz test.** a** Urine color after the addition of 4-dimethylaminobenzaldehyde; **b** Urine color after the addition of first reagent and chloroform; to the left of the figure is patient’s sample and to the right is the control sample

**Fig. 2:**
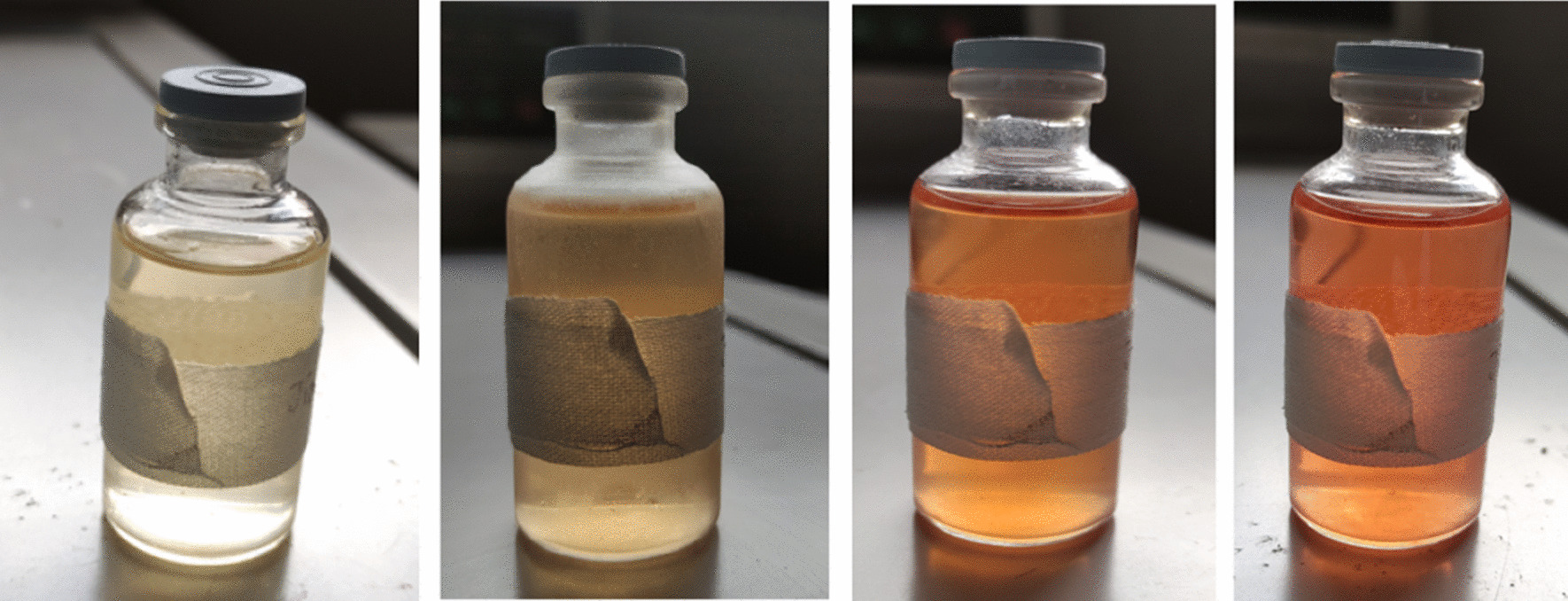
Changes in urine color on exposure to light (Day 1, Day 3, Day 5, Day 7)

The patient's clinical presentation, including severe abdominal pain, was not relieved even with morphine. After all these thorough investigations, severe uncorrected hyponatremia, severe body ache especially in the lower limbs, and precipitation of the symptoms following fasting ushered our conjectures toward the diagnosis of porphyria-related disorders. With the suspicion of porphyria, a urine sample was sent to Biochemistry Laboratory. Watson-Schwartz test was positive (Fig. 1); hence, it indicated the presence of porphobilinogen in urine substantiating the diagnosis of acute porphyria crisis. The urine color also darkened on exposure to light (Fig. 2). The symptoms were gradually relieved with the consumption of carbohydrate-rich foods. Genetic confirmation of HMBS mutation was not possible due to financial constraints. However, on the grounds of a strong clinical picture and screening for PBG in urine, a final diagnosis of acute intermittent porphyria was made.

She was kept on a high-sugar diet as hemin was unavailable. Gradually her pain subsided, her blood pressure came to normal and her bowel habit also improved. Pregabalin was given for neuropathic pain, which was gradually tapered and stopped during follow-up. The patient was counseled to avoid fasting. On follow-up examination in the outpatient department (OPD), she was asymptomatic with normal blood pressure and bowel habit.

## Discussion and conclusion

The prevalence of acute intermittent porphyria (AIP) is estimated to be 5–10 cases per 100,000 persons [6]. The disease is preponderant in females as compared to males and symptoms show up usually in adolescence [7]. Despite an inherited disorder, about 90% of patients who inherit the disease may be asymptomatic for a lifetime which marks the diagnostic challenge of the disease [8]. AIP is not diagnosed over the shallow examination, rather it is a finding of exception after a series of expensive and time-consuming investigations, which can often go futile. The common clinical presentation of AIP includes altered mentation, and unexplained abdominal pain, chest and back pain [9]. Apart from a single characteristic symptom like severe unresolving abdominal pain, other supportive histories for acute porphyria diagnosis include dark/reddish urine, new-onset hypertension, hyponatremia, proximal muscle weakness, pain associated with luteal phase in the menstrual cycle, use of exacerbating drugs and low carbohydrate diets. Behavioral changes like irritability, hypertension and tachycardia are conundrums that dupe porphyria diagnosis [5].

The clinical presentation of porphyria patients is baffling due to overlapping neurovisceral and systemic manifestations. Acute porphyrias are mostly presented with neurologic manifestations including neuropathic abdominal pain, peripheral neuropathy, and mental disturbances. Any impediment in the diagnosis of an acute porphyric attack may further aggravate the neurological symptoms inciting irreversible damage. Failure to identify neurovisceral symptoms like muscle weaknesses at an early stage may cause the patient to succumb to respiratory muscle paralysis, an important complication of porphyria which is life-threatening [10, 11]. Timely diagnosis is a subject of clinical acuity in the management of porphyria [12], which also aids in the exemption of alternative diagnosis.

The first-line treatment options for seizures include phenytoin, valproate, benzodiazepines, and lacosamide, among which many are porphyrinogenic [13]. The porphyrinogenic anti-seizure drugs are metabolized by cytochrome p450 enzymes in the liver that leads to increased heme synthesis, which results in increased heme precursors further provoking the porphyria crises [14]. Management of seizures with medications possessing porphyrinogenic potential, unaware of the porphyric status of the patient, can trigger acute attacks [15].

Neurological complications mostly comprise peripheral neuropathy, acute encephalopathy, and acute symptomatic seizures [9]. Seizures in patients with porphyria may be precipitated by hyponatremia due to the syndrome of inappropriate antidiuretic hormone [16]. Our patient presented with non-specific symptoms of porphyria upon admission but a gradual aggravation of electrolyte imbalance. Hyponatremia during an acute attack is also the presenting symptom in our case. These conditions may arise due to hypothalamic involvement, inappropriate antidiuretic hormone secretion, excess gastrointestinal and renal sodium loss, vomiting, and resuscitation with large volumes of intravenous dextrose solution [5, 17]. The cardiac manifestations may be due to increased catecholamine production [5].

The clinical presentation of our case was similar to an adolescent boy in Nepal as described by Dhital *et al.* in which the patient dimly recalled precipitation of acute abdomen after the episodes of fasting and low-calorie intake [12]. Fasting induces proliferator-activated receptor γ coactivator 1α (PGC-1α) in the liver that turns on the the expression of *ALAS-1* gene [18]. Hence, uncontrolled upregulation of ALA synthase enzyme leading to the accumulation of heme precursors causes precipitation of acute porphyria attack in conditions of low-calorie intake [19].

Other common presenting symptoms include gastrointestinal complications (abdominal pain, vomiting, constipation, diarrhea), cardiovascular complications (tachycardia, systemic arterial hypertension, heart palpitations), and hematological complications(anemia) [4, 5]. Anemic presentation is mostly seen in cutaneous porphyria, congenital erythropoietic porphyria, and less commonly, hepatoerythropoietic porphyria [20]. Acute hepatic porphyria(AHP) should be considered in the differential diagnosis of unexplained abdominal pain. Screening for porphyria when symptoms are suggestive, is easily accessible and prompt, however they are not included in the comprehensive workup for investigations in practice [21]. Urine porphobilinogen and porphyrin normalized to creatinine is a recommended first-line testing for AHP [22].

Hemin is used for the therapeutic management of porphyria but owing to its high cost and reduced availability in our context, we could not administer it. However, the “glucose effect” is the commonly used mechanism as an alternative for management. Considerate carbohydrate inclusion in diet checks the ALA synthase activity reducing heme precursors [21, 23].

Porphyrias are still the concern of diagnostic dilemma making them highly underdiagnosed. Diagnostic delay can often cause the patients to undergo critical surgical procedures like appendectomies or cholecystectomies with writhing agony, multiple investigations, unnecessary treatment burden, and prolonged hospital stay. Inappropriate treatment of porphyria may incite severe life-threatening conditions like paresis and even death. Driving the diagnostic protocol toward porphyria-related disorders require high clinical suspicion. This disease can be approached beforehand using simple screening tools where available to curtail any dreadful consequences.

## Supplementary Information

**Additional file 1.** Laboratory Parameters not included in the text. **Table S1.** Routine parameters on admission. **Table S2.** Serial electrolyte analysis on different days. **Table S3:** Urine Biochemistry(spot)

## Data Availability

This published article includes all the required data.

## Abbreviations

ABG: Arterial blood gas
AHP: Acute hepatic porphyria
AIP: Acute intermittent porphyria
ALA-S: 5’-Aminolevulinic acid synthase
ANA: Anti-nuclear antibody
ARR: Aldosterone renin ratio
CNS: Central nervous system
CT: Computed tomography
GCS: Glasgow Coma Scale
HMBS: Hydroxymethylbilane synthase
ICU: Intensive Care Unit
PGC-1α: Proliferator-activated Receptor γ Coactivator 1α
PICU: Paediatric Intensive Care Unit
RAS: Renal artery stenosis
SAIO: Sub-acute Intestinal Obstruction
TUTH: Tribhuvan University Teaching Hospital
