# Supplementary material for: Porphyria: a case report
Source: J Med Case Rep. 2022 Dec 28;16:490. doi: 10.1186/s13256-022-03708-w (PMC9795741; doi:10.1186/s13256-022-03708-w)
Supplement: Supplementary file 1 — Additional file 1. Laboratory Parameters not included in the text. Table S1. Routine parameters on admission. Table S2. Serial electrolyte analysis on different days. Table S3: Urine Biochemistry(spot) [file 13256_2022_3708_MOESM1_ESM.docx]

**Additional file 1**

**Laboratory Parameters not included in the text:**

Serum ADA was 41.2 U/L (<30.0 U/L), Erythrocyte Sedimentation Rate (ESR) was 39 mm/hr (0 – 9 mm/hr), Serum ACTH was 5.7 pg/ml (5 – 60 pg/ml), C-Reactive protein (CRP) was 2.75 mg/L (<6.0 mg/L), Serum Cortisol 14 µg/ml (2.9 – 17.3 µg/ml). TFT was normal. Urine spot chloride was 144 mEq/L (110 – 250 mEq/L). She tested negative for SARS-CoV2 PCR.

With a diagnostic lab test of hyponatremia (116 mEq/L), a 3% NaCl infusion was started. Other symptomatic management for treatment used were: Amlod (5 mg), Ketorolac (30 mg), Ondem (4 mg), Atenolol (25 mg), Tramadol (50 mg), Lactulose syrup, MgSO4 (40 mg/ml), Buscopan (30 mg), Fentanyl, Pregabalin (50 mg), Vitamin B12 (1500 mg).

Renal and liver function tests were normal.

Abdominal and pelvic CT scans showed prominent ascending and transverse colon with collapsed bowel distal to the splenic flexure. CT scan of the head showed minimal bilateral maxillary sinusitis while other findings were normal. The magnesium level fluctuated, ranging from 1.1 to 2.4 mg/dl.

**Table S1: Routine parameters on admission:**

| Parameter | Value | Unit | Reference Range |
| --- | --- | --- | --- |
| Haematology | | | |
| TLC | 10200 | /cmm | 4000 – 11000 |
| DLC | 81.6 – 11.4 – 6.0 – 1.0 | % |  |
| Hb | 10.9 | gm/dl | 11.6 – 15 |
| PCV | 32 | % | 36 – 54 |
| Platelet | 231000 | /cmm | 150000 - 450000 |
| RBC | 3.79 | million/cmm | 4.0 – 5.5 |
| MCV | 84.43 | fl | 82 – 100 |
| MCH | 28.76 | pg | 27 – 32 |
| MCHC | 34.06 | % | 32 – 36 |
| RDW-CV | 12.3 | % | 11 – 16 |
| Biochemistry | | | |
| Glucose (Random) | 4.8 | mmol/L | 3.8 – 7.8 |
| Urea | 4.4 | mmol/L | 2.8 – 7.0 |
| Creatinine | 52.0 | µmol/L | 40 – 110 |
| Sodium | 116.0 | mEq/L | 135 – 145 |
| Potassium | 3.7 | mEq/L | 3.5 – 5.1 |
| Total Bilirubin | 19.0 | µmol/L | 3 – 21 |
| Direct Bilirubin | 5.0 | µmol/L | 0 – 5 |
| GPT | 27.0 | U/L | 5 – 45 |
| GOT | 32.0 | U/L | 5 – 40 |
| ALP | 81.0 | U/L | < 306 |
| Total Protein | 68.0 | g/L | 60 – 80 |
| Albumin | 37.0 | g/L | 37 – 47 |

Hyponatremia, hypokalemia, and hypomagnesemia was the presenting complication in the patient. The serial electrolyte analysis on different days is as follows:

**Table S2: Serial electrolyte analysis on different days**

| Date | Sodium (mEq/L) | Potassium (mEq/L) | Magnesium(mg/dl) | Calcium (mg/dl) |
| --- | --- | --- | --- | --- |
| 2022/08/10 | 121 | 2.8 |  |  |
| 2022/08/11 (07:30) | 124 | 3.4 |  |  |
| 2022/08/11 (13:30) |  | 3.2 | 1.5 | 8.6 |
| 2022/08/11 (20:00) |  | 3.7 | 3.4 |  |
| 2022/08/12 | 122 | 3.9 |  |  |
| 2022/08/13 | 132 | 3.2 |  |  |
| 2022/08/14 | 133 | 3.1 | 2.1 |  |
| 2022/08/15 |  | 3.6 |  |  |
| 2022/08/16 | 133 | 3.7 | 1.26 |  |
| 2022/08/17 |  | 3.4 |  |  |
| 2022/08/18 |  | 3.8 | 1.7 |  |
| 2022/08/19 |  | 3.9 |  |  |
| 2022/08/20 (12:20) | 116.0 | 3.7 |  |  |
| 2022/08/20 (22:24) | 120.0 | 3.8 |  |  |
| 2022/08/21 |  |  | 1.4 |  |
| 2022/08/22 | 113.0 | 3.1 |  |  |
| 2022/08/23 | 109.0 | 3.1 |  |  |
| 2022/08/24 | 117.0 | 3.6 |  |  |
| 2022/08/25 | 126.0 | 3.5 |  |  |
| 2022/08/26 | 125.0 | 3.4 | 1.2 | 8.0 |
| 2022/08/28 | 123.0 | 3.8 | 1.5 |  |
| 2022/08/30 | 127.0 | 3.7 |  |  |
| 2022/09/02 | 130.0 | 4.3 |  |  |
| 2022/09/04 | 135.0 | 3.9 |  |  |

**Table S3: Urine Biochemistry(spot)**

| Date | Sodium (mEq/L) | Potassium (mEq/L) | Osmolarity (mOsm/L) |
| --- | --- | --- | --- |
| 2022/08/11 | 169.0 |  | 371.0 |
| 2022/08/24 | 114.0 | 16.0 | 323.7 |
